# Identification of Shared Genes and Pathways in Periodontitis and Type 2 Diabetes by Bioinformatics Analysis

**DOI:** 10.3389/fendo.2021.724278

**Published:** 2022-01-25

**Authors:** Junho Kang, Eun Jung Kwon, Mihyang Ha, Hansong Lee, Yeuni Yu, Ji Wan Kang, Yeongjoo Kim, Eun Young Lee, Ji-Young Joo, Hye Jin Heo, Eun Kyoung Kim, Tae Woo Kim, Yun Hak Kim, Hae Ryoun Park

**Affiliations:** ^1^ Medical Research Institute, Pusan National University, Busan, South Korea; ^2^ Interdisciplinary Program of Genomic Data Science, Pusan National University, Busan, South Korea; ^3^ Department of Oral Pathology, School of Dentistry, Pusan National University, Yangsan, South Korea; ^4^ Department of Periodontology, School of Dentistry, Pusan National University, Yangsan, South Korea; ^5^ Departmment of Anatomy, School of Medicine, Pusan National University, Yangsan, South Korea; ^6^ Department of Orthopaedic Surgery, Pusan National University Yangsan Hospital, School of Medicine, Pusan National University, Yangsan, South Korea; ^7^ Department of Biomedical Informatics, School of Medicine, Pusan National University, Yangsan, South Korea

**Keywords:** differentially expressed genes, hub genes, gene expression omnibus, biomarker, Fc gamma receptor

## Abstract

**Introduction:**

It is well known that the presence of diabetes significantly affects the progression of periodontitis and that periodontitis has negative effects on diabetes and diabetes-related complications. Although this two-way relationship between type 2 diabetes and periodontitis could be understood through experimental and clinical studies, information on common genetic factors would be more useful for the understanding of both diseases and the development of treatment strategies.

**Materials and Methods:**

Gene expression data for periodontitis and type 2 diabetes were obtained from the Gene Expression Omnibus database. After preprocessing of data to reduce heterogeneity, differentially expressed genes (DEGs) between disease and normal tissue were identified using a linear regression model package. Gene ontology and Kyoto encyclopedia of genes and genome pathway enrichment analyses were conducted using R package ‘*vsn*’. A protein-protein interaction network was constructed using the search tool for the retrieval of the interacting genes database. We used molecular complex detection for optimal module selection. CytoHubba was used to identify the highest linkage hub gene in the network.

**Results:**

We identified 152 commonly DEGs, including 125 upregulated and 27 downregulated genes. Through common DEGs, we constructed a protein-protein interaction and identified highly connected hub genes. The hub genes were up-regulated in both diseases and were most significantly enriched in the Fc gamma R-mediated phagocytosis pathway.

**Discussion:**

We have identified three up-regulated genes involved in Fc gamma receptor-mediated phagocytosis, and these genes could be potential therapeutic targets in patients with periodontitis and type 2 diabetes.

## Introduction

Periodontitis (PD) and diabetes are complex chronic diseases, and the link between the two diseases has been continuously reported (1, 2). Previous epidemiologic studies have reported that the risk of periodontitis increases by about three times in diabetics compared to non-diabetic patients. In addition, adults with HbA1c levels above 9% had a higher prevalence of severe periodontitis than adults without diabetes (3). These epidemiological studies indicate that depending on the state of diabetes, it is associated with an increase in the prevalence and severity of periodontitis. The study that suggested the possibility of diabetes incidence according to periodontal disease status was a study by the Gila River Indian Community, a Native American population with a high prevalence of diabetes (4). In this study, patients with untreated severe periodontitis were associated with poor glycemic control (HbA1c >9.0%, 75 mmol/mol), suggesting that diabetes control may be inhibited. Thus, the identification of factors about common pathogenic changes of them might provide an important clue for the treatment.

PD is one of the most representative chronic inflammatory diseases, which is caused by periodontopathogenic bacteria harbored in a gingival crevice, whereas type 2 diabetes (T2DM) is a metabolic disease with complicated pathogenic factors. Thus, the diseases are independent of each other with respect to disease classification, but several studies have shown that the two diseases share risk factors such as socioeconomic status, smoking, and age (5). Although understanding environmental causes, such as demographic factors may play an important role in the prevention and resolution of diseases, clarification of specific pathogenic determinants beyond simple demographic factors is needed to develop a strategy for curing the two diseases effectively. Studies have suggested the importance of inflammation as a linking factor, based on the clinical findings of a decrease in HbA1c levels after surgical and/or non-surgical periodontal therapy and the resultant alleviation of PD (6). The problem is that inflammation is not a particular etiological agent but a generic term for a pathological process comprising complicated processes. Although numerous cytokines that are involved in the inflammatory process could be suggested as major factors in both diseases, these may be simply modifying factors that could modulate the pathogenesis of the diseases. Thus, clinical and/or experimental studies have limitations in clearly revealing the common pathogenic mechanisms.

Microarray technology provides systematic biological solutions from hardware to software systems. Simultaneously scans the hybridization signal of tens of thousands of gene probes on the chip and enables quantitative analysis of the transcript profile of the sample (7–9). Microarray analysis can identify biomarkers for disease and provide insight into novel therapeutic targets. The wide application of microarray analysis has generated large amounts of data, most of which are already stored in public databases such as GEO. Integrating and analyzing this data can lead to novel and important clues about the disease (10). In recent years, several independent studies have performed microarray analyzes of periodontitis and diabetes (11, 12). In independent studies, due to the heterogeneity of tissues or samples, most microarray results were limited or inconsistent, and were generated in a single cohort study. To minimize these issues, we tried to identify potential biomarkers of periodontitis and diabetes by integrating expression data from several independent cohorts through bioinformatics methods.

## Materials and Methods

### Data Acquisition

**Figure 1:**
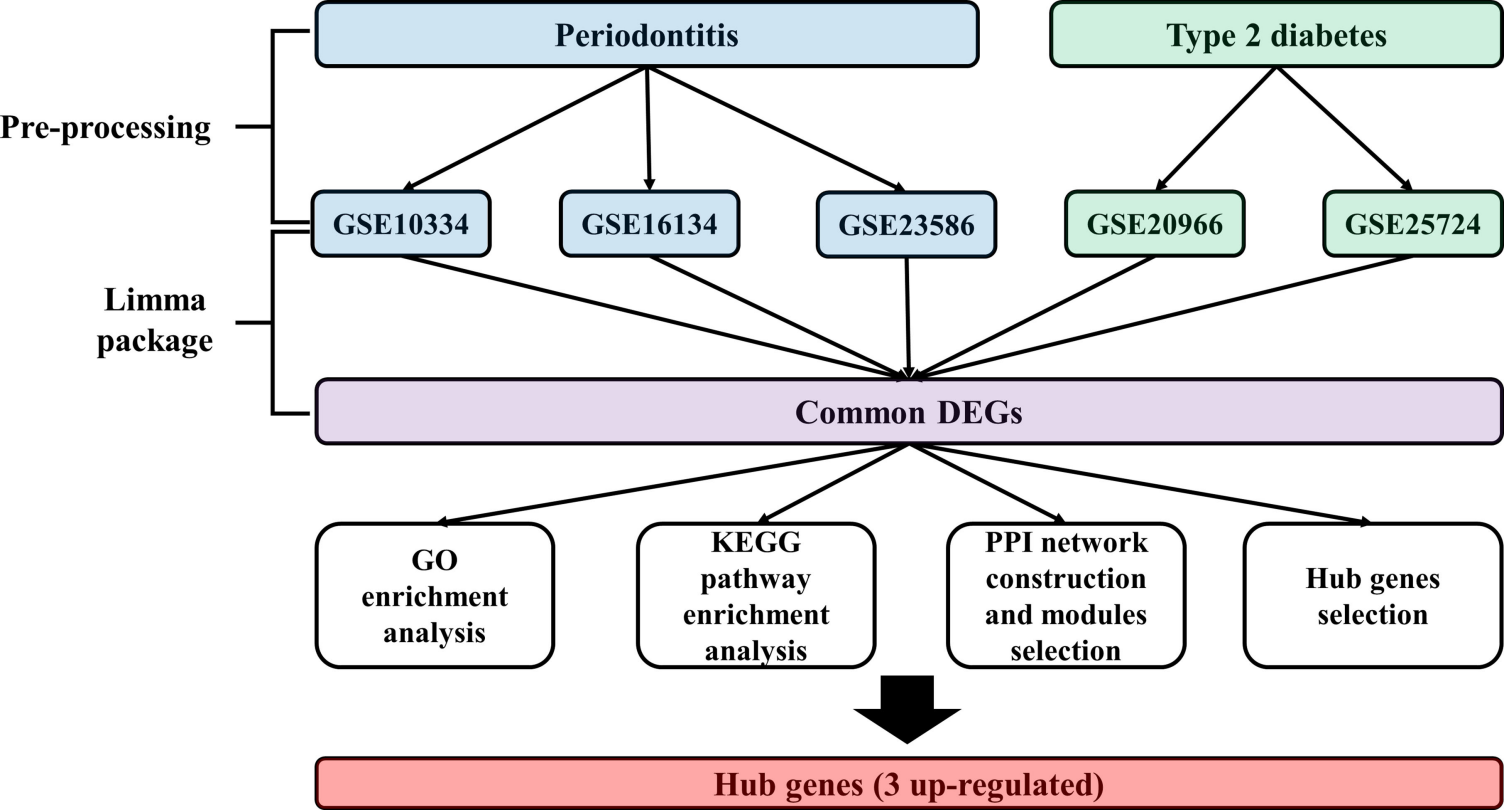
Study flow chart.

Expression profiling data by array of the PD and T2DM were obtained from the Gene Expression Omnibus (GEO) database (13). The accession numbers were GSE10334 (12), GSE16134 (14), GSE23586 (15), GSE20966 (11), and GSE25724 (16). The keywords used to select the datasets are shown in **Table 1**. All included datasets were related to gene expression data from disease (PD, T2DM) groups and/or normal groups and were downloaded for differentially expressed genes (DEG) analysis. The flow chart of this study is shown in **Figure 1** and the characteristics of the included datasets are listed in **Table 2**.

**Table 1:** Electric search strategy in GEO.

Database	GEO	
Disease	Periodontitis	Type 2 diabetes mellitus
**Search** **Term**	[(“Periodontitis” (MeSH Terms) AND “Homosapiens”(porgn: txid9606)]	[(“diabetes mellitus, type 2”(MeSH Terms) OR type 2 diabetes (All Fields)] AND [“pancreas” (MeSH Terms) OR pancreatic (All Fields)] AND [“tissues”(MeSH Terms) OR tissue (All Fields)] AND “Homo sapiens”(porgn) AND [“gds”(Filter) AND “Expression profiling by array”(Filter)].
**Filter**	Expression profiling by array Human	Expression profiling by array Human
**Results**	19	4

**Table 2:** Characteristics of included datasets.

	GSE10334		GSE16134		GSE23586	
	Normal	Periodontitis	Normal	Periodontitis	Normal	Periodontitis
Type of tissue/cell	Gingival tissue		Gingival tissue		Gingival tissue	
Platform	Affymetrix Human Genome U133 Plus 2.0 Array		Affymetrix Human Genome U133 Plus 2.0 Array		Affymetrix Human Genome U133 Plus 2.0 Array	
Sample size	64	183	69	241	3	3
Age (mean)	42.0		39.3		35.0	58.0
Gender						
(Male: Female)	5:5		5:5		8:6	7:7
Pocket depth (mm)	1 ~ 4	5 ~ 11	3.9 ± 0.7		1.6 ± 0.2	6.5 ± 2.0
References	Demmer et al. (12)		Kebschull et al. (14)		Abe et al. (15)	
	**GSE20966**			**GSE25724**		
	**Normal**		**T2DM**	**Normal**		**T2DM**
Type of tissue/cell	Beta-cells			Pancreatic islet		
Platform	Affymetrix Human X3P Array			Affymetrix Human Genome U133A Array		
Sample size	10		10	7		6
Gender						
(Male: Female)	6: 4		7: 3	4: 33: 3		
Age (year)	60.3 ± 3.0		67.3 ± 4.3	58.1 ± 12.8		70.5 ± 7.3
BMI	30.6 ± 3.2		30.9 ± 4.0	24.8 ± 1.9		26.0 ± 1.8
References	Marselli et al. (11)			Dominguez et al. (16)		

### Data Preprocessing

There is inevitable heterogeneity between studies published in GEO. To minimize heterogeneity, we pre-processed raw data using the same method and only included studies conducted on the same platform. Briefly, data preprocessing (background correction and normalization) for raw probe intensities was performed using the “*vsn*” (variance stabilizing normalization, v 3.24.0) R/Bioconductor package (17). Based on the annotation file downloaded from GEO, the probe id was converted to a genetic symbol. When multiple probes corresponded to a single gene, the average level of the probes was calculated as the expression value of the gene. In addition, probes that did not correspond to the genetic symbols were removed. To screen the intersectional genes significantly expressed in each cohort, R package “*venn*” was used to plot Venn diagrams.

### Differential Expression Analysis

To identify DEGs between disease and normal tissue, we performed the linear regression model package, “limma”, in R language (v 3.42.2) (18). We considered genes with adjusted p-value < 0.05 to be significantly differentially expressed. The method used for adjusted p-value is false discovery rate. The log_2_ fold change > 0.5 was set to upregulated genes, and log_2_ fold change < 0.5 was set to downregulated genes.

### Functional and Pathway Enrichment Analysis

Gene ontology (GO) and Kyoto Encyclopedia of Genes and Genome (KEGG) pathway enrichment analyses were performed using R package “clusterProfiler” (v 3.14.3) (19). The common DEGs identified in each disease were included in GO term enrichment analysis and KEGG pathway enrichment analysis, which were performed separately for up and downregulated genes. Terms with ≥ 5 associated genes and ≥ 2 DEGs in each experiment were included; FDR adjustment of p-values was performed using the Benjamini-Hochberg method.

### PPI Network Construction and Module Selection

Protein–protein interaction (PPI) network was constructed using the Search Tool for the Retrieval of Interacting Genes (STRING) database (20) to identify the relevant pathways and functions of common DEGs. The minimum required interaction score used to construct the PPI network was 0.4. We used molecular complex detection (MCODE) (21) for optimal module selection. The parameters used in MCODE were as follows: degree cutoff, 2; cluster finding, node score cutoff, 0.2; k-core, 2; and max.depth, 100. CytoHubba (22) was used to identify the highest linkage hub gene in the network. The parameters used in CytoHubba were as follows: the top 10 nodes ranked: degree, display options: display the expanded subnetwork.

### Receiver Operating Characteristic (ROC) Curves Analysis

To confirm the diagnostic performance of hub genes, ROC curves were analyzed. The package used for the analysis was the r package, pROC (v1.18.0).

### Experimental Validation of Hub Genes

For experimental validation, we recruited samples from 5 normal, 9 patients with periodontitis, and 4 patients with periodontitis with diabetes. Inclusion criteria included individuals aged 20 years or older. Exclusion criteria included subjects with current or previous history of significant systemic diseases, such as cardiovascular accidents, cancer, or renal failure. Patients with a prior history of using non-steroidal anti-inflammatory drugs, antibiotics or blood thinner were also excluded. Periodontal condition of subjects was assessed, then moderate or severe periodontitis patients were selected for periodontitis group. Periodontal conditions are according to the classification established in 1999 International Workshop of the American Academy of Periodontology (APP) for a Classification of Periodontal Diseases and Conditions. The presence of diabetes was determined by the value of Hb1Ac, and periodontitis patients showing more than 6.5% Hb1Ac were grouped into periodontitis with diabetes mellitus. Controls exhibited healthy periodontal tissue of less than 2 mm probing depth and no inflammatory signs such as gingival swelling, redness, and bleeding on probing. Briefly, tissue samples were obtained from periodontal pockets which were affected by periodontitis. Tissues of healthy controls without periodontitis were harvested from gingiva during gingivectomy or crown lengthening. Characteristics of clinical samples used for experimental validation is shown in **Supplementary Table 1**.

### Quantitative Real-Time Polymerase Chain Reaction

This study was conducted in accordance with the Declaration of Helsinki and was approved by the Ethics Committee of Pusan National University Dental Hospital (IRB: PNUDH 2020-032). Total RNA was extracted using the gingival tissues from healthy control, periodontitis patients and periodontitis patients with diabetes. Complementary DNA (cDNA) was synthesized using the Smart Gene Compact cDNA Synthesis kit (Smart Gene, South Korea). Quantitative real-time PCR was performed using the QuantStudio™ 3 Real-Time PCR System (Applied Biosystems, USA). Target mRNA expression relative to housekeeping gene expression (GAPDH) was calculated using the delta-delta Ct (ΔΔCT) method. A list of primers used in the quantitative real-time polymerase chain reaction is shown in **Supplementary Table 2**.

### Statistical Analysis

We performed one-way ANOVA to check the differences between each group, and Tukey’s multiple comparison test was used for the *post-hoc* test. P values less than 0.05 were considered significant. All statistical analyses were performed using R (v4.0.5).

## Results

### Identification of Common DEGs for Each Disease

**Figure 2:**
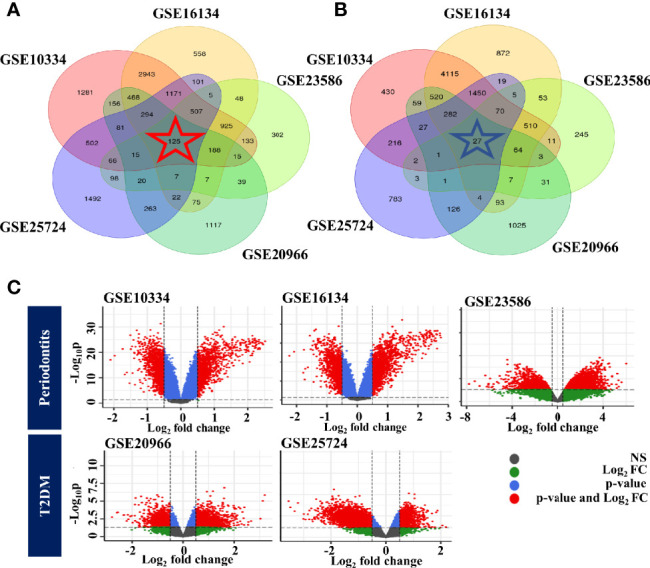
Identification of common DEGs for each disease. **(A)** Venn diagram of upregulated genes. **(B)** Venn diagram of downregulated genes. **(C)** Volcano plot of DEGs identified in individual cohorts. The color of the points in the volcano plot indicates gray: not significant, green: passed only the log2 fold change, blue: passed only the p-value, red: passed the p-value and log2 fold change. PD, Below: T2DM.

First, background correction and normalization of all expressed microarray data sets were performed to proceed with the DEG analysis. We identified DEGs in individual cohorts. We then identified overlapping genes among DEGs from individual cohorts. As a result, we identified 125 up-regulated and 27 down-regulated genes. Venn diagrams and volcano plots of DEGs identified in individual cohorts are shown in **Figure 2**.

### Functional Enrichment Analysis of Common DEGs

GO and KEGG pathway analyses were performed to confirm the function of 152 common DEGs (**Supplementary Table 3**). The top 10 significantly enriched GO terms of the up-regulated and down-regulated genes are shown in **Supplementary Figure 1**, and the results of KEGG pathway analysis are shown in **Supplementary Figure 2**. The results of GO, KEGG analyses in each disease were described in **Supplementary Table 3**.

### PPI Network Construction, Module Selection, and Identification of Hub Genes

**Figure 3:**
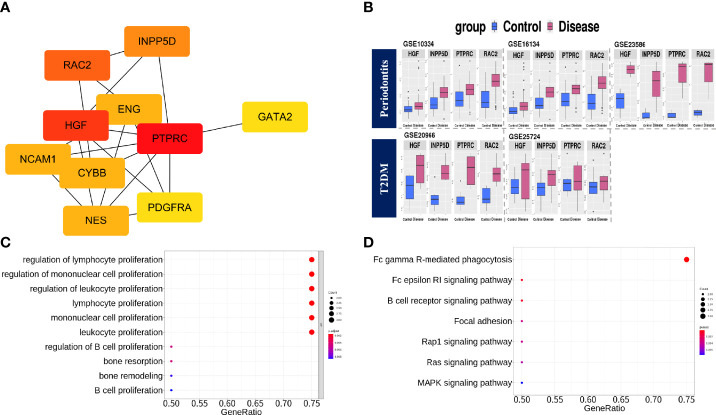
PPI network construction, module selection, and identification of hub genes. **(A)** The top 10 highly connected genes based on degree score. **(B)** Boxplots of expression values in the individual cohorts of hub genes. Blue: Control group, Red: Disease group. **(C)** The significantly enriched BP terms of GO analysis with hub genes. The meaning of the dot color is as follows: red: adjusted p-value < 0.002, purple: < 0.004, blue < 0.008. **(D)** KEGG pathway analysis using hub genes. The meaning of the dot color is as follows: red: adjusted p-value < 0.002, purple: < 0.004, blue < 0.006.

To identify the protein–protein interactions of common DEGs, we used the STRING network-based protein interaction assay to create a PPI network. The most significant module consisted of 41 nodes and 93 edges. We identified highly connected genes in the most significantly constructed modules, and *PTPRC*, *HGF*, *RAC2*, *INPP5D*, *ENG*, *NES*, *CYBB*, *NCAM1*, *PDGFRA*, and *GATA2* were the top 10 highly connected genes (**Figure 3**). We then defined genes with a degree score of 10 or higher as hub genes. The hub genes identified in this study were *PTPRC*, *HGF*, *RAC2*, and *INPP5D*. The expression values of the hub genes in individual cohorts are shown in **Figure 3**, and the characteristics are shown in **Supplementary Table 4**. We performed functional enrichment analysis of the hub genes. In GO-terms, the negative regulation of interleukin-6 production and regulation of B cell proliferation were significantly enriched (adjusted p < 0.05, **Figure 3**). In the KEGG pathway, the Fc gamma R-mediated phagocytosis and the Fc epsilon RI signaling pathway were significantly enriched (adjusted p < 0.05, **Figure 3**).

### Identification of Diagnostic Performance of Hub Genes in Periodontitis and Type 2 Diabetes

ROC analysis was performed using the included GEO datasets and clinical samples to evaluate the diagnostic performance of each hub gene in T2DM and PD. GSE10334 dataset, and their AUC values (RAC2:0.863, INPP5D:0.818, PTPRC:0.749, HGF:0.697) were showed in **Supplementary Figure 3A**. GSE16134 dataset, and their AUC values (RAC2:0.890, INPP5D:0.836, PTPRC: 0.785, HGF:0.722) were showed in **Supplementary Figure 3B**. GSE23586 dataset, and their AUC values (RAC2:1.000, INPP5D:0.889, PTPRC:1.000, HGF:1.000) were showed in **Supplementary Figure 3C**. GSE20966 dataset, and their AUC values(RAC2:0.620, INPP5D:0.880, PTPRC:0.710, HGF:0.630) were showed in **Supplementary Figure 3D**. GSE25724 dataset, and their AUC values (RAC2:0.952, INPP5D:0.952, PTPRC:0.952, HGF:0.786) were showed in **Supplementary Figure 3E**. In periodontitis samples, and their AUC values (RAC2:0.956, INPP5D:1.000, PTPRC:1.000, HGF:0.733) were shown in **Supplementary Figure 4**. In periodontitis patients with diabetes samples, and their AUC values (RAC2:0.900, INPP5D:0.850, PTPRC:0.850, HGF:0.450) were showed in **Supplementary Figure 5**. These results suggest that all three hub genes except HGF have good diagnostic performance.

### Validation of the Hub Genes in Clinical Samples

**Figure 4:**
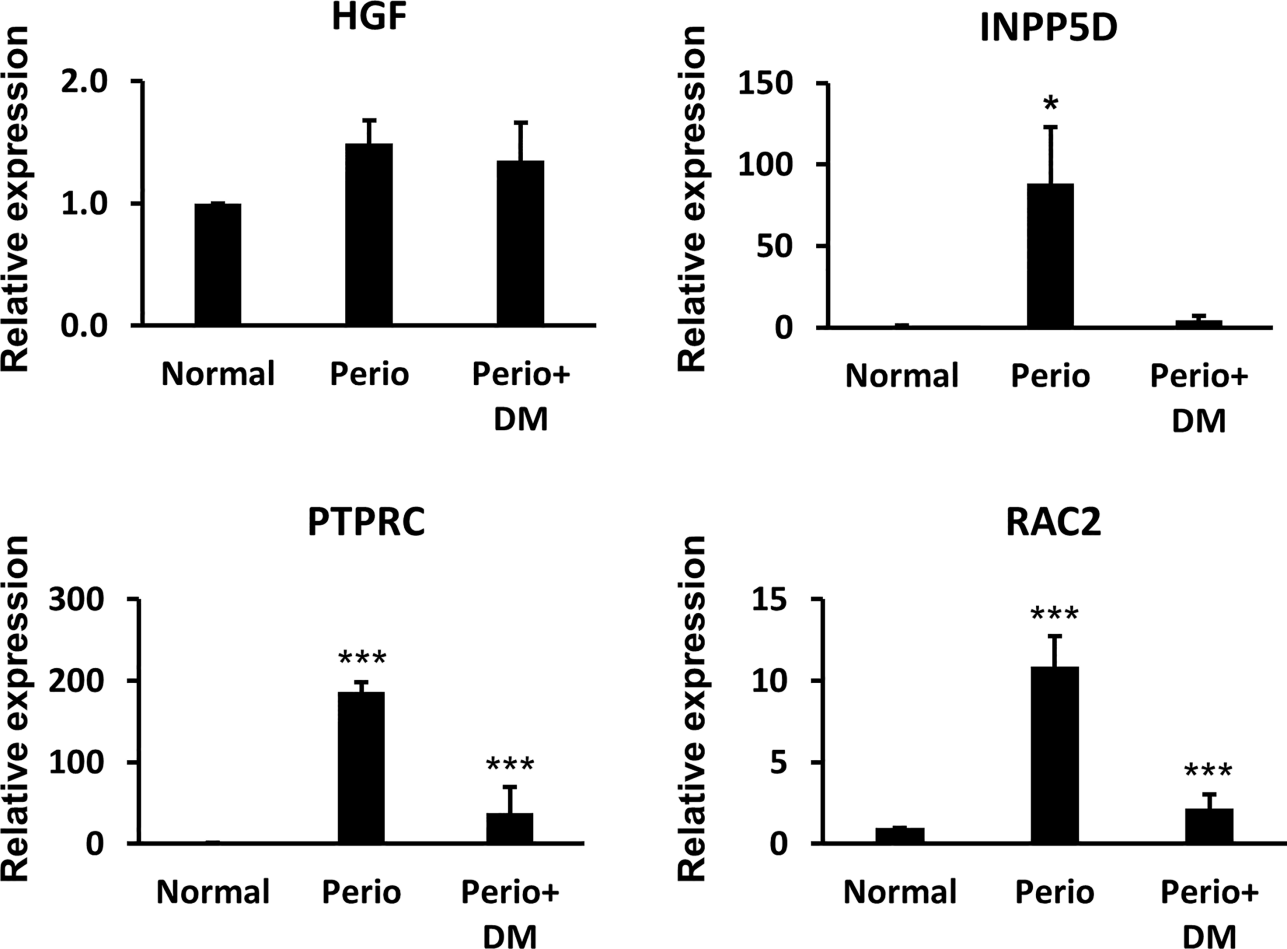
The results of Quantitative reverse transcription-PCR between healthy controls, periodontitis patients, and periodontitis patients with diabetes. *p < 0.05, ***p < 0.005.

We performed a Real-Time qPCR assay using gingival tissues from healthy controls, periodontitis patients, and periodontitis patients with diabetes to experimentally validate the hub gene. As a result, there was no significant difference in HGF between the groups, and INPP5D was significantly increased only in the periodontitis patient group (p <0.05). RAC2 and PTPRC were significantly increased in the periodontitis (p<0.005) and periodontitis patients with T2DM group (p <0.005) compared with the healthy control group. Real-Time qPCR results are shown in **Figure 4**.

## Discussion

Recently, studies on the molecular pathogenesis of diseases using bioinformatics tools have emerged in the field of biomedicine. Such studies have revealed numerous findings on crucial factors that are important for the development and progression of the disease as well as for linking genes between various diseases. PD and diabetes are chronic diseases with a complex mechanism and a bidirectional relationship (23). The risk of PD is known to increase by two to three times in people with diabetes (24) and the level of glycemic control is key in determining the risk (25). To date, research on PD and diabetes has mainly focused on T2DM, but studies have shown that type 1 diabetes is also associated with periodontal destruction (26). These previous findings indicate that there may be a potential common key gene that causes both PD and diabetes.

In this study, we identified 152 common DEGs, including 125 up-regulated genes and 27 down-regulated genes. 152 common DEGs were significantly enriched in focal adhesion, cAMP signaling pathway, and Fc gamma R-mediated phagocytosis signaling pathway, which are known to be associated with immune response. Focal adhesion is known to up-regulate several adhesion molecules due to the activation of endothelial cells in the inflammatory response and trigger the interaction of these cells with white blood cells (27). cAMP regulates a number of key pathways that affect the immune system and, depending on the cell type, can trigger both pro-inflammatory and anti-inflammatory effects. It is known that poor regulation of this signaling can affect the pathogenesis of inflammatory skin diseases such as atopic dermatitis and psoriasis (28). Fc gamma R mediated phagocytosis controls humoral and innate immunity essential for response to infection and chronic inflammation (29). These results indirectly indicate that the signaling pathways that control various inflammations are involved in both chronic inflammatory diseases. However, we tried to identify potential target genes or signaling pathways that could be more important of these signaling pathways. Therefore, we identified highly connected hub genes among 152 common DEGs and tried to find signaling pathways containing these genes. As a result, the highly linked hub genes were *PTPRC*, *RAC2*, *HGF*, and *INPP5D*. In functional enrichment analysis of these genes, it was the most significantly enriched Fc gamma R mediated phagocytosis, and *PTPRC*, *RAC2*, *INPP5D* hub genes included in this signaling pathway were thought to be potential therapeutic targets.

Protein tyrosine phosphatase, receptor type, C (PTPRC) plays a key role in the signaling regulation of integrin and cytokine receptors, lymphocyte development, and antigen receptor signaling (30). Moreover, it differentially regulates Fc-gamma R-mediated tyrosine phosphorylation in polymorphonuclear neutrophil activation (31). In diabetes, protein tyrosine phosphatase is known to act as a negative regulator of insulin signaling, and expression of PTPRC is associated with residual β-cell function in type 1 diabetes (32). In addition, there is sufficient evidence that PTPRC acts as an important regulator of immune cell function, protection against viral infection, and is associated with immunodeficiency and viral susceptibility (1, 33). These previous findings and our findings support the hypothesis that upregulation of PTPRC regulates viral susceptibility and immune cell function and contributes to the pathogenesis of PD and diabetes. *RAC2* encodes a small GTPase of guanosine triphosphate (GTP) metabolic protein. Genetic mutations in *RAC2* cause severe phagocytic immunodeficiency characterized by severe infection in infancy (34). There are three isoforms of RAC, and *RAC1* and *RAC2* are differentially activated. Previous studies have shown decreased NADPH oxidase activation and IgG-mediated phagocytosis in neutrophils in *Rac2*-/- mice (35). In addition, NADPH oxidase preferentially interacts with *RAC2* (36). *RAC2* is a T1D candidate gene located at the 22q12.3 chromosomal risk locus (37). Functional knockdown experiments of *Rac2* showed that *Rac2* knockdown increases cytokine-induced apoptosis (38). Moreover, *Rac2*-deficient mice are much more susceptible to PD than WT controls, but the IL-17 response has not been determined. Nonetheless, mice deficient in *Rac2* display abundant mononuclear cell infiltration in the junctional epithelium and gingival connective tissue (39). *INPP5D*, also known as *SHIP1*, is an enzyme with phosphatase activity. Loss of *Inpp5d* function in mice causes both macrophages and B lymphocytes to become hypersensitive to stimuli (40). *INPP5D* partially attenuates BCR signaling through its association with Fc gamma receptor IIB and acts as an effector for other inhibitory receptors in numerous immune cell types (41). Small molecule inhibitors of *INPP5D* are known to improve obesity and the metabolic syndrome related to aging and diet (42). The *RAC2* and *INPP5D* genes are still insufficiently studied in both chronic diseases, but given the role revealed in previous studies, the upregulation of both genes could be a novel biomarker for chronic hyperglycemia.

Fc gamma R-mediated phagocytosis occurs when the Fc gamma receptor of monocyte-macrophages or neutrophils binds to IgG (43). The Fc gamma receptor is an essential participant in many immune system effector functions, such as the release of inflammatory mediators, antibody-dependent cytotoxicity, and phagocytosis (44, 45). Recent studies have confirmed that Fc gamma receptor glycan modifications are also important for interactions with antibodies and downstream immune responses (46, 47). Additionally, these previous studies suggest a role in inflammatory diseases and potential new therapeutic targets. Our findings support previous findings. Therefore, we believe that three hub genes that are commonly upregulated in both chronic diseases may be potential therapeutic targets.

There are several limitations to our study, and they are as follows: 1. In the included studies, information on various factors related to the disease was not provided and thus could not be evaluated. 2. Although we tried to minimize the heterogeneity of expression values according to the platform, only the microarray studies were included, so genes with low expression values may have been excluded. 3. Although the hub genes were experimentally validated through clinical samples, heterogeneity exists in the results because they were obtained from gingival tissue from PD patients with T2DM, not from pancreatic tissue.

## Conclusions

The three hub genes identified in our study are involved in immune response regulation and insulin resistance and are all involved in the Fc gamma R mediated phagocytosis signaling pathway. In addition, previous studies have suggested an important role of the Fc gamma receptor in chronic inflammatory diseases and as a new therapeutic target. Therefore, we believe that up-regulation of these three genes affects two chronic diseases and can be potential therapeutic targets. Finally, we believe that further validation through experiments on these genes is also necessary.

## Data Availability Statement

The original contributions presented in the study are included in the article/**Supplementary Material**. Further inquiries can be directed to the corresponding authors.

## Author Contributions

KJ: conceptualization, data analysis, data curation, and writing original draft. KEJ: conceptualization, data analysis, and data curation. HM: data curation. LH: investigation. YY: investigation. KJW: investigation. KY: investigation. LEY: methodology and investigation. JJY: methodology and investigation. HHJ: experimental validation. KEK: experimental validation. KTW: experimental validation, writing review. KYH: supervision, writing review, and editing. PHR: supervision, writing review, and editing. All authors contributed to the article and approved the submitted version.

## Funding

This work was supported by the National Research Foundation of Korea (2020R1A2C1005203, 2020R1C1C1003741, and NRF-2018R1A5A2023879).

## Conflict of Interest

The authors declare that the research was conducted in the absence of any commercial or financial relationships that could be construed as a potential conflict of interest.

## Publisher’s Note

All claims expressed in this article are solely those of the authors and do not necessarily represent those of their affiliated organizations, or those of the publisher, the editors and the reviewers. Any product that may be evaluated in this article, or claim that may be made by its manufacturer, is not guaranteed or endorsed by the publisher.

## Supplementary Material

Supplementary Figure 1 The results of GO analysis using common DEGs. The color of the dots indicates the p-value and size is the number of included genes. The meaning of the dot color is as follows: red: adjusted p-value < 0.002, purple: < 0.004, blue < 0.008. **(A)** upregulated genes **(B)** downregulated genes

Supplementary Figure 2 The results of KEGG pathway analysis using common DEGs. The color of the dots indicates p-value and size is the numbers of included genes. The meaning of the dot color is as follows: red: adjusted p-value < 0.002, purple: < 0.004, blue < 0.006. A: upregulated genes B: downregulated gene.

Supplementary Figure 3 The results of ROC analysis for hub genes in included gene expression omnibus datasets. **(A)** GSE10334, **(B)** GSE16134, **(C)** GSE23586, **(D)** GSE20966, **(E)** GSE25724.

Supplementary Figure 4 The results of ROC analysis for hub genes in periodontitis patient samples. **(A)** RAC2, **(B)** INPP5D, **(C)** PTPRC, **(D)** HGF.

Supplementary Figure 5 The results of ROC analysis for hub genes in periodontitis with diabetes patient samples. **(A)** RAC2, **(B)** INPP5D, **(C)** PTPRC, **(D)** HGF.

Supplementary Table 3 The results of GO and KEGG pathway analyses.
