# Supplementary figures and images for: Identification of Shared Genes and Pathways in Periodontitis and Type 2 Diabetes by Bioinformatics Analysis
Source: Front Endocrinol (Lausanne). 2022 Jan 25;12:724278. doi: 10.3389/fendo.2021.724278 (PMC8822582; doi:10.3389/fendo.2021.724278)

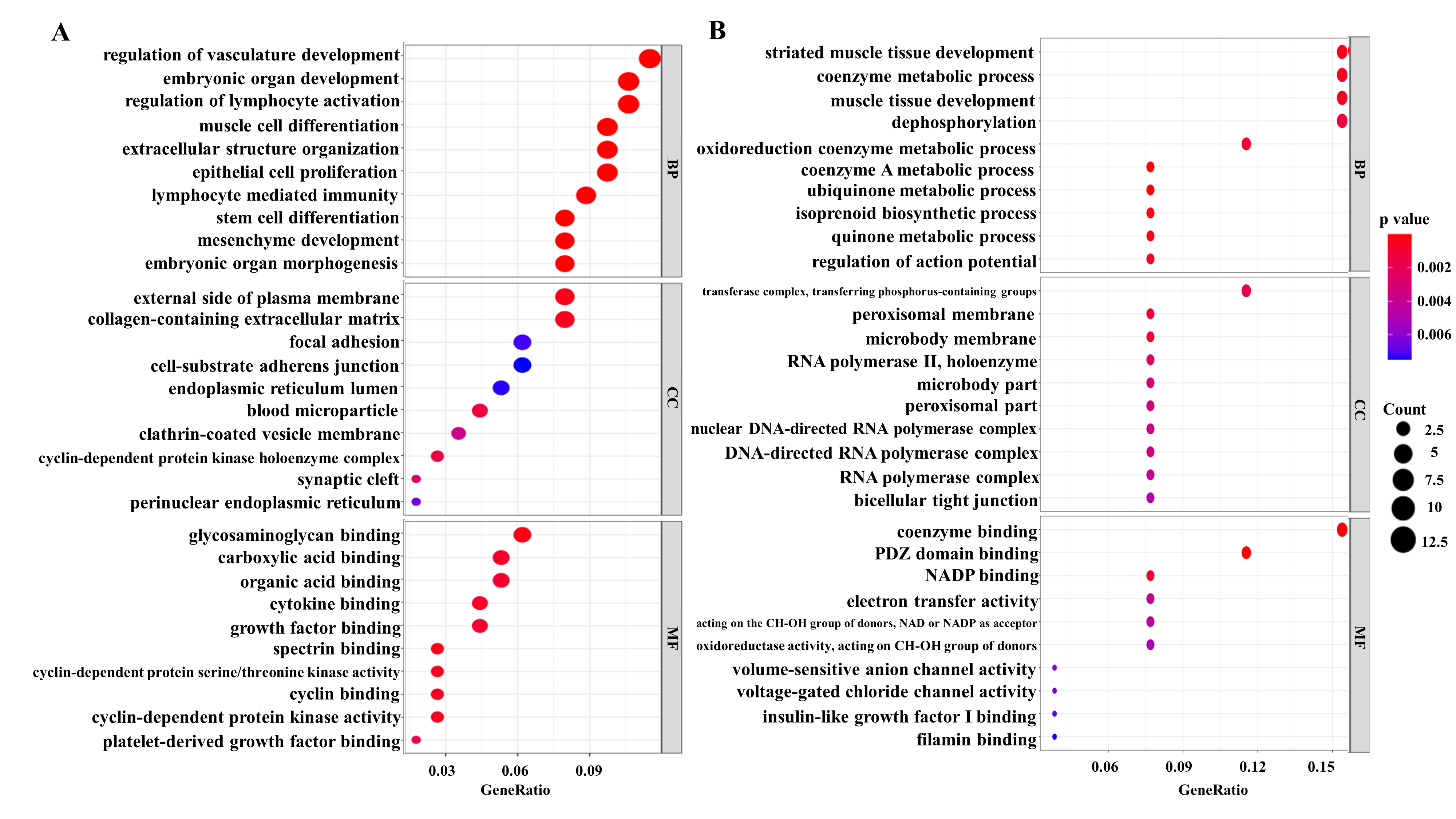

Supplement: Supplementary Figure 1 — The results of GO analysis using common DEGs. The color of the dots indicates the p-value and size is the number of included genes. The meaning of the dot color is as follows: red: adjusted p-value < 0.002, purple: < 0.004, blue < 0.008. (A) upregulated genes (B) downregulated genes [file Image_1.tif]

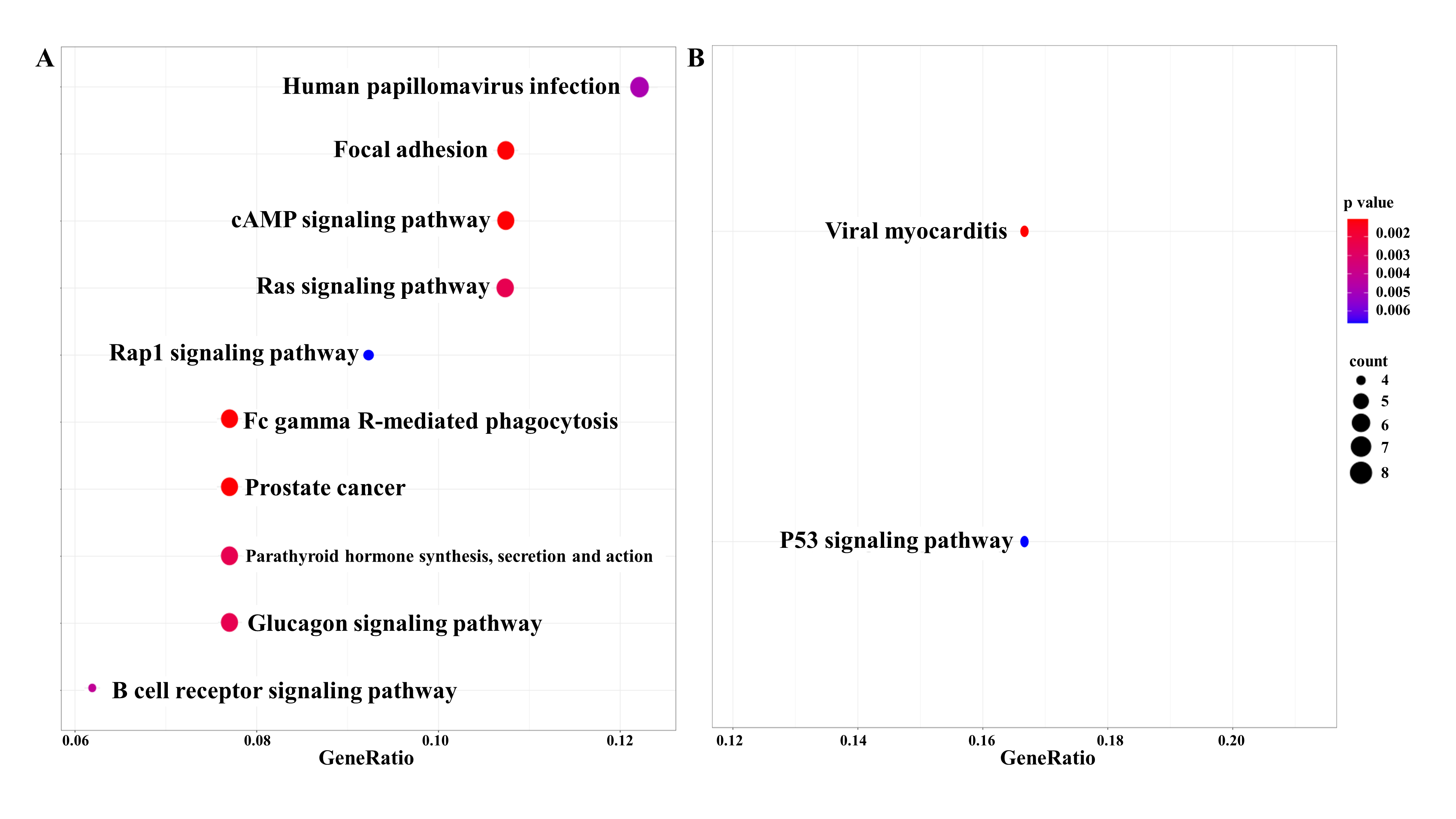

Supplement: Supplementary Figure 2 — The results of KEGG pathway analysis using common DEGs. The color of the dots indicates p-value and size is the numbers of included genes. The meaning of the dot color is as follows: red: adjusted p-value < 0.002, purple: < 0.004, blue < 0.006. A: upregulated genes B: downregulated gene. [file Image_2.tif]

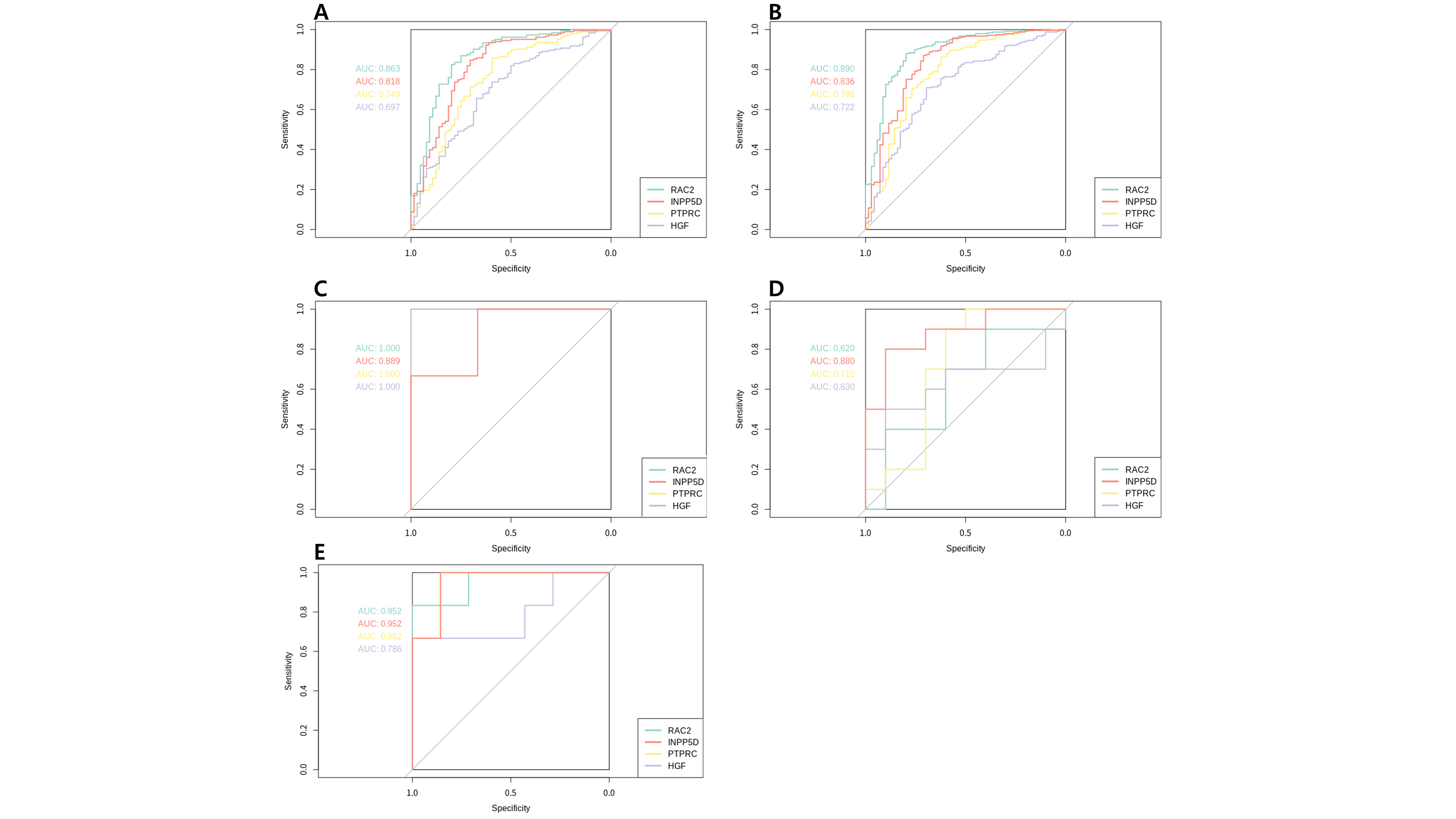

Supplement: Supplementary Figure 3 — The results of ROC analysis for hub genes in included gene expression omnibus datasets. (A) GSE10334, (B) GSE16134, (C) GSE23586, (D) GSE20966, (E) GSE25724. [file Image_3.tif]

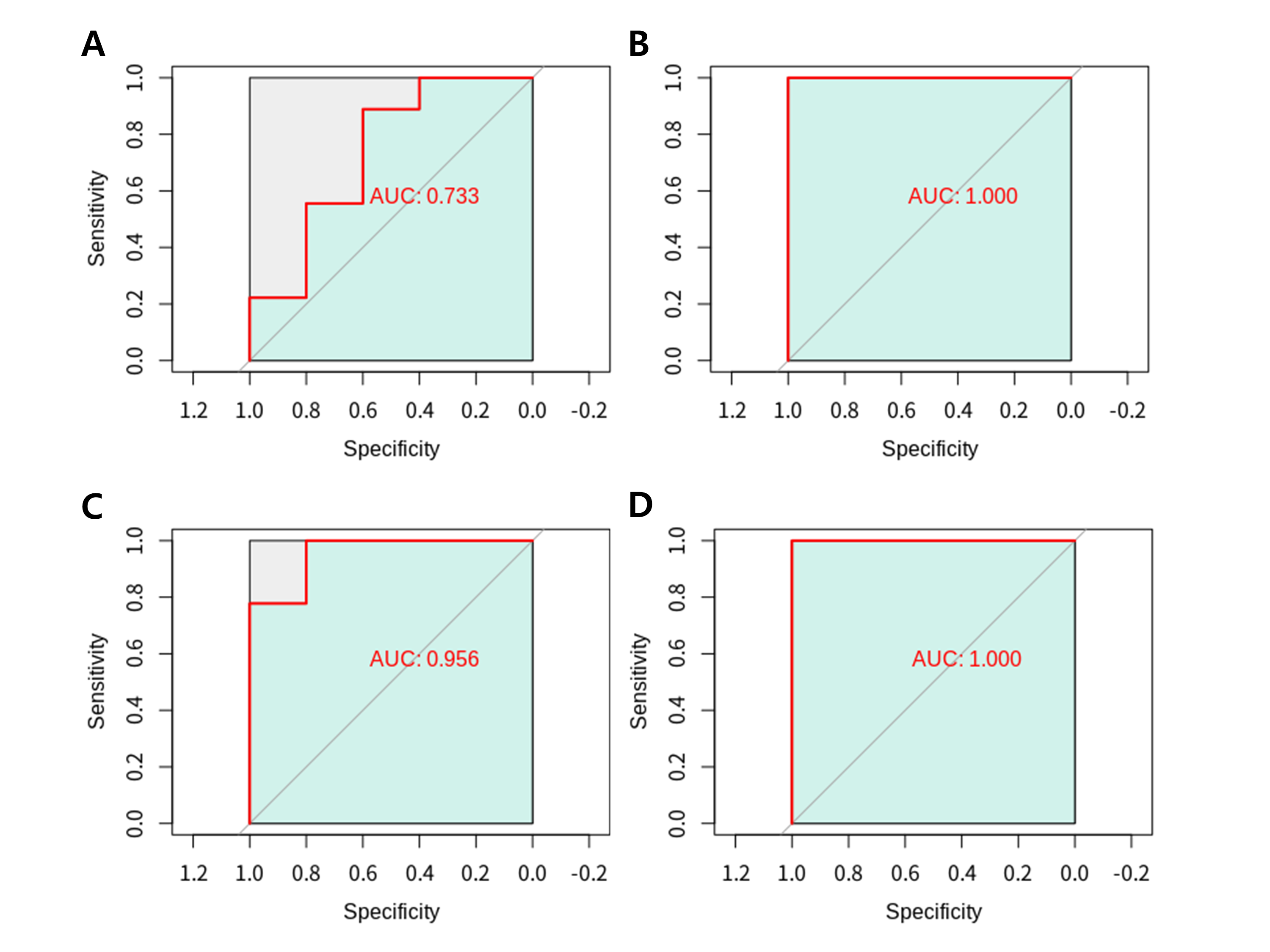

Supplement: Supplementary Figure 4 — The results of ROC analysis for hub genes in periodontitis patient samples. (A) RAC2, (B) INPP5D, (C) PTPRC, (D) HGF. [file Image_4.tif]

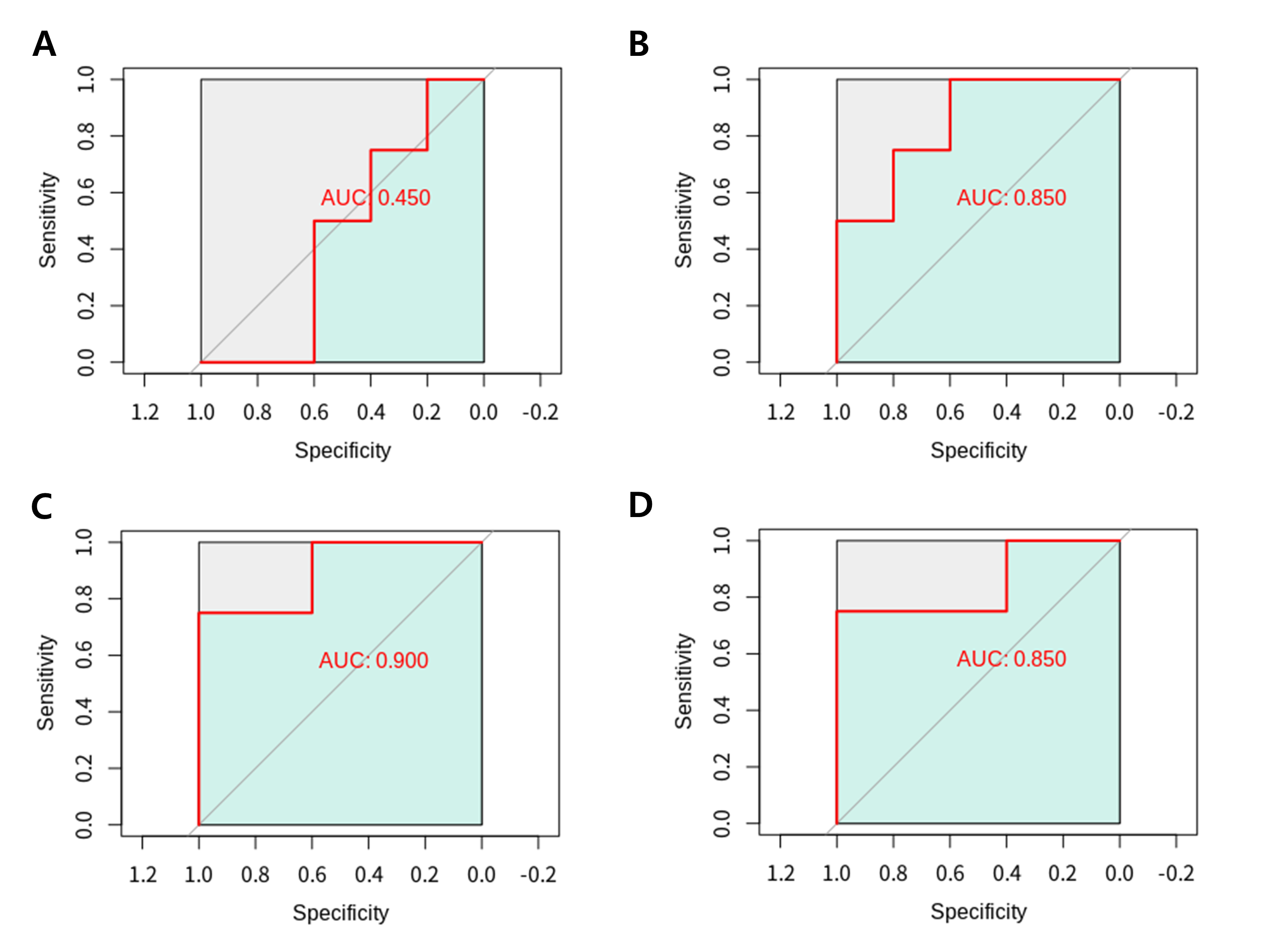

Supplement: Supplementary Figure 5 — The results of ROC analysis for hub genes in periodontitis with diabetes patient samples. (A) RAC2, (B) INPP5D, (C) PTPRC, (D) HGF. [file Image_5.tif]
