# Supplementary material for: Identification of Shared Genes and Pathways in Periodontitis and Type 2 Diabetes by Bioinformatics Analysis
Source: Front Endocrinol (Lausanne). 2022 Jan 25;12:724278. doi: 10.3389/fendo.2021.724278 (PMC8822582; doi:10.3389/fendo.2021.724278)
Supplement: Supplementary file 6 [file Table_1.docx]

|  | **Normal** | **Periodontitis** | | **Periodontitis with diabetes** | |
| --- | --- | --- | --- | --- | --- |
| Sample size | 5 | 9 | | 4 | |
| Age (mean) | 57 | 49 | | 66 | |
| Gender  (Male:Female) | 1:4 | 2:7 | 3:1 | |  |
| Periodontitis severity  (Moderate:Severe) | - | 4:5 | 1:3 | |  |
| Type of  tissue | Periodontal tissue | Periodontal tissue | | Periodontal tissue | |

*Supplementary table 1. Characteristics of clinical samples used for experimental validation*

*Supplementary table 2. List of primers used for Quantitative real-time polymerase chain reaction.*

| Gene | Forward primer (5′-3′) | Reverse primer (5′-3′) |
| --- | --- | --- |
| Human HGF | atg tcc tcc tgc atc tcc tcc t | atg cta ttg aag ggg aac cag agg |
| Human INPP5D | tcg acg ttg agt ctg gga aac tga | ctt gga gag aaa cca gga cgt gat |
| Human PTPRC | acc tgg aat ccc cct caa aga tca | gag gcc tac act tga cat gca tac |
| Human RAC2 | ttc tca tca gct aca cca cca acg | cct tca gtt tct cga tgg tgt cct |
| Human GAPDH | cat gtt cgt cat ggg gtg aac ca | agt gat ggc atg gac tgt ggt cat |

*Supplementary table 4. The characteristics of hub genes.*

| **Symbols** | **Score** | **Descriptions** | **Functions** |
| --- | --- | --- | --- |
| *PTPRC* | 21 | Receptor-type tyrosine-protein phosphatase C 2 | It is a protein tyrosine-protein phosphatase required for T-cell activation through antigen receptors and acts as a positive regulator of T-cell co-activation when binding to DPP4^14^ . |
| *HGF* | 13 | Hepatocyte growth factor | Potent mitogen for mature parenchymal hepatocyte cells, seems to be a hepatotrophic factor, and acts as a growth factor for a broad spectrum of tissues and cell types^14^ . |
| *RAC2* | 11 | Ras-related C3 botulinum toxin substrate 2 | It is a plasma membrane-associated small GTPase that circulates between an active GTP-binding state and an inactive GDP-binding state, and in its active state binds to various effector proteins to regulate cellular responses such as secretory processes, phagocytosis of apoptotic cells, and epithelial cell polarization^14^. |
| *INPP5D* | 10 | Phosphatidylinositol 3,4,5-trisphosphate 5-phosphatase 1 | Phosphatidylinositol (PtdIns) phosphatase that specifically hydrolyzes the 5-phosphate of phosphatidylinositol- 3,4,5-trisphosphate (PtdIns(3,4,5)P3) to produce PtdIns(3,4)P2, thereby negatively regulating the PI3K (phosphoinositide 3-kinase) pathways and acts as a negative regulator of B-cell antigen receptor signaling^14^. |
